# Analysis of the role of the human papillomavirus 16/18 E7 protein assay in screening for cervical intraepithelial neoplasia: a case control study

**DOI:** 10.1186/s12885-020-07483-z

**Published:** 2020-10-14

**Authors:** Linghua Kong, Xiaoping Xiao, Huiping Lou, Pengfei Liu, Shuhui Song, Moudu Liu, Tao Xu, Ying Zhang, Caijuan Li, Ruoli Guan, Yan Li, Xin Yu, Haiyuan Liu, Qingbo Fan, Honghui Shi, Lan Zhu

**Affiliations:** 1 Department of Obstetrics and Gynecology, Peking Union Medical College Hospital, Chinese Academy of Medical Sciences, Shuaifuyuan No. 1, Dongcheng District, Beijing, 100730 China; 2 Department of Health Sciences, Peking Union Medical College Hospital, Chinese Academy of Medical Sciences, Shuaifuyuan No. 1, Dongcheng District, Beijing, 100730 China; 3 AMID Biotech (Tianjin) Co., Ltd., No. 4, Haitai Development 2nd Road, Binhai Gaoxin District, Tianjin, 300450 China; 4 Institute of Basic Medical Sciences, Peking Union Medical College, Chinese Academy of Medical Sciences, 5 Dong Dan San Tiao, Beijing, 100005 China

**Keywords:** Cervical intraepithelial neoplasia (CIN), E7 oncoprotein, High-risk human papillomavirus (Hr-HPV)

## Abstract

**Background:**

Cervical cancer is the second-most common gynecological cancer, early screening plays a key role in the diagnosis and treatment of cervical intraepithelial neoplasia (CIN). Sustained E7 protein expression is the pathological basis for CIN and cervical cancer.

**Methods:**

We collected the cervical cell samples of women who visited the gynecological clinic of Peking Union Medical College Hospital between September 2018 and September 2019 and submitted them to the high-risk human papillomavirus (Hr-HPV) test. We performed a magnetic particle–based chemiluminescence enzyme immunoassay to analyze the HPV16/18 E7 protein level in CIN of different severities and compared the results with those of cervical pathology (gold standard) and the HPV test.

**Results:**

The positive rate of HPV16/18 E7 protein increased with the severity of CIN: 26.6% in normal tissue, 58.3% in CIN1, and 70.6% in CIN2 or higher (CIN2+). For CIN2+, the sensitivity, specificity, positive predictive value (PPV), and negative predictive value (NPV) of the E7 protein were 70.6, 67.9, 52.2, and 82.3%, respectively. These values of the HPV test were 86.8, 44.5, 43.7, and 87.1%, respectively. With the combination of the E7 protein assay and HPV test, the specificity for diagnosing CIN2+ was 78.1%, which was significantly higher than that of the HPV test alone.

**Conclusions:**

HPV16/18 E7 protein level is correlated with the severity of CIN and has a high concordance rate with the pathological result. For cervical cancer screening, the combination of HPV16/18 E7 protein assay and HPV test improves the CIN diagnostic specificity, detection rate, and detection accuracy.

## Background

Cervical cancer is the second-most common gynecological cancer [1]. Early screening plays a key role in the diagnosis and treatment of cervical intraepithelial neoplasia (CIN). Cervical cytology is the first screening technique and is widely used. The sensitivity and specificity of cytology are 53.0 and 96.5%, respectively [2]. To further improve the diagnostic sensitivity and address limitations such as the shortage and varying skill levels of technicians, in 2014 the World Health Organization (WHO) recommended the use of a high-risk human papillomavirus (Hr-HPV) test for cervical cancer screening in developing countries [3, 4]. However, approximately 90% of HPV infections are transient [3]. Different studies have shown great variability in the specificity of HPV testing for high-grade CIN, from 24.8 to 56.1% [5, 6]. Researchers agree that the high sensitivity and low specificity of the HPV test contribute to a high colposcopy referral rate [7]. Improving the diagnostic specificity would prevent unnecessary panic and reduce colposcopy referral rates and healthcare costs. To achieve this, it is important to search for more effective biomarkers. HPV E7 protein inhibits retinoblastoma protein, thereby promoting the uncontrolled proliferation and malignant transformation of infected cells [8, 9]. Sustained E7 protein expression is the pathological basis for CIN and cervical cancer [10–13]. Therefore, E7 protein may be a marker for CIN [14, 15]. In this study, we analyzed HPV 16/18 E7 protein level in CIN of different severities in order to investigate the relationship between the results of the HPV 16/18 E7 protein assay and the severity of CIN, as well as to investigate the value of the HPV16/18 E7 protein assay in CIN screening and evaluated its role as an auxiliary diagnostic biomarker.

## Method

### Clinical data

**Fig. 1:**
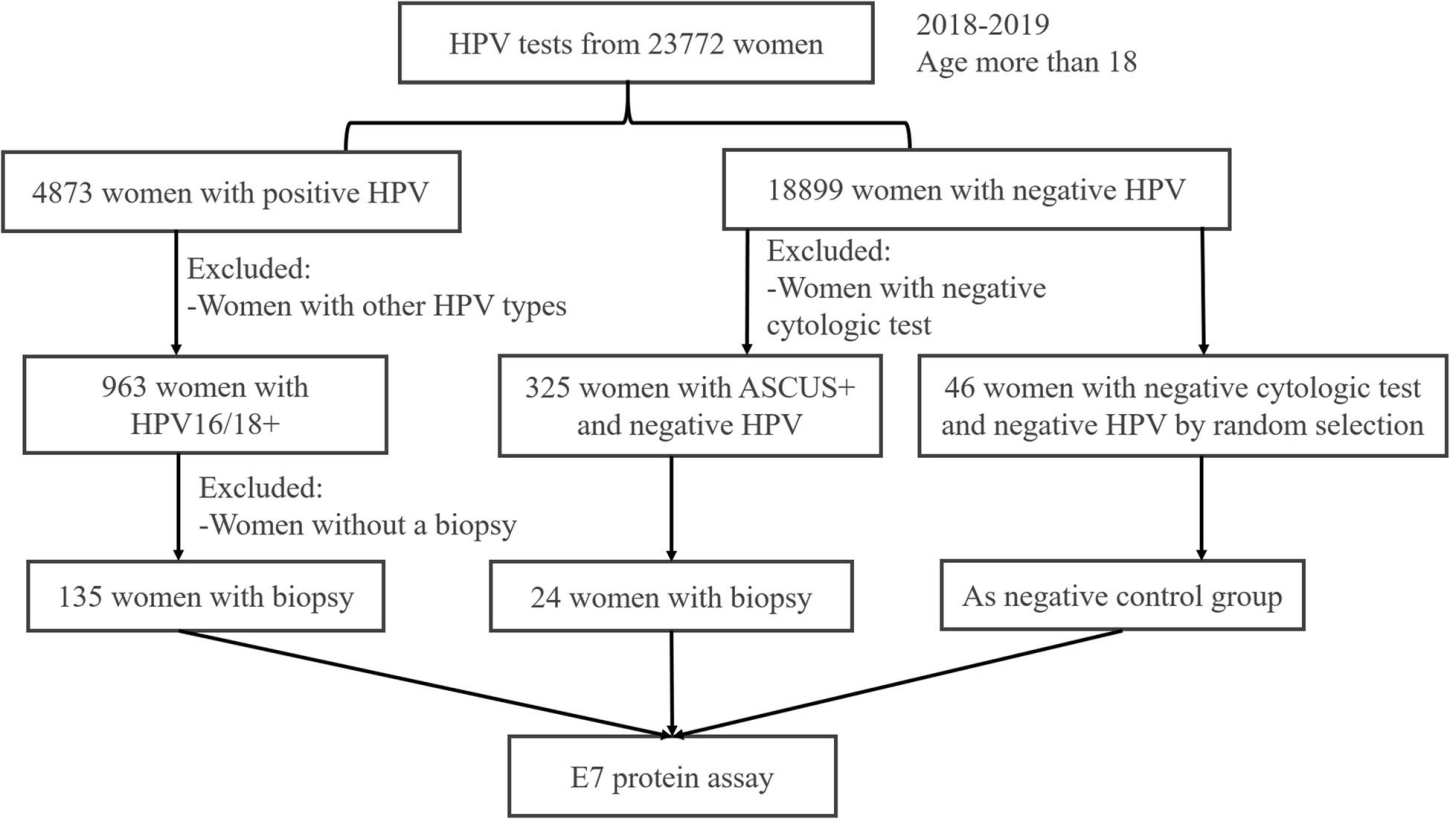
The flow chart of the study

The study was approved by the Ethics Committee of Peking Union Medical College Hospital (approval number HS1624) and was conducted after obtaining the patient’s informed consent. Cervical cell samples of 23,772 women aged 18 or above who visited the gynecological clinic of Peking Union Medical College Hospital between September 2018 and September 2019 were collected and submitted to the HPV test. A total of 963 samples with HPV16/18 positive and 325 HPV negative patients with remarkable cytological abnormalities (Atypical Squamous Cells of Undetermined Significance and more serious, ASC-US+) were included in the study. Moreover, a total of 159 patients underwent colposcopy and biopsy for pathological examination, among which, 135 patients were positive for HPV16/18 and 24 samples were ASC-US+ with HPV negative. Besides, 46 randomly selected samples who did not undergo colposcopy or biopsy due to negative HPV test and cytologic test were included in the negative control group for statistical analysis (Fig. 1). The patients were aged from 24 to 79 (42.57 ± 11.27) years.

### The aim

To investigate the relationship between the results of the HPV 16/18 E7 protein assay and the severity of CIN, as well as to investigate the value of the HPV16/18 E7 protein assay in CIN screening.

### The design

We collected the cervical cell samples of 23,772 women who visited the gynecological clinic and submitted them to the HPV test. A total of 135 samples with HPV16/18 positive and histological results and 24 samples with ASC-US+, HPV negative, and histological results were included in the study. Moreover, 46 randomly selected samples that were negative for both HPV and cytologic test were included in the negative control group for statistical analysis. We performed a magnetic particle–based chemiluminescence enzyme immunoassay to analyze the HPV16/18 E7 protein level in CIN of different severities and compared the results with those of cervical pathology (gold standard) and the HPV test.

### Cytologic test

Cytologic specimens were processed with the ThinPrep liquid-based cytology systems (Hologic, CA, USA). Cytology results were classified with the Bethesda System for Reporting Cervical Cytology as revised in 2014 [16].

### Hr-HPV DNA test

The Cobas 4800 HPV test (Roche Diagnostics, GmbH, Mannheim, Germany), which is a qualitative test for detection of HPV DNA, was used to analyze the samples. This test amplifies target DNA in cervical epithelial cells by PCR and nucleic acid hybridization (Cobas PCR collection media, Roche Molecular Systems, Inc.) to detect 14 Hr-HPV types, of which HPV 16 and HPV 18 are of the greatest importance. This analysis also enables the detection of Hr-HPV types 31, 33, 35, 39, 45, 51, 52, 56, 58, 59, 66, and 68 at clinically significant levels of infectivity.

### E7 protein assay

The HPV16/18 E7 magnetic particle–based chemiluminescence enzyme immunoassay kit from AMID Biotech (Tianjin) Co., Ltd. was used to analyze the E7 protein level according to the instructions. The patients were instructed to refrain from intercourse and vaginal medication. The supernatant was collected for analysis within 2 h. The assay used the double-antibody sandwich method, where magnetic particles were used as the solid phase of the immune response, and highly specific antibodies were used to detect high-risk E7 protein in cervical cell samples.

### Pathological examination

The pathological result was used as the gold standard. CIN was pathologically classified as mild (CIN1), moderate (CIN2), or severe (CIN3).

### Statistical analysis

SPSS v25.0 was used for statistical analysis. Count data are expressed as a percentage or n (%). The sensitivity, specificity, positive predictive value (PPV), and negative predictive value (NPV) were evaluated with the receiver operating characteristic (ROC) curve and area under the curve (AUC). *P* < 0.05 was considered statistically significant.

## Results

In this study, 46 patients were negative on both the cytologic test and HPV test. Pathologically, 67 patients were normal, 24 patients were CIN1, and 68 patients were CIN2/CIN3. E7 protein level was analyzed in 205 cell samples.

### Diagnostic efficacy of ROC curve

**Fig. 2:**
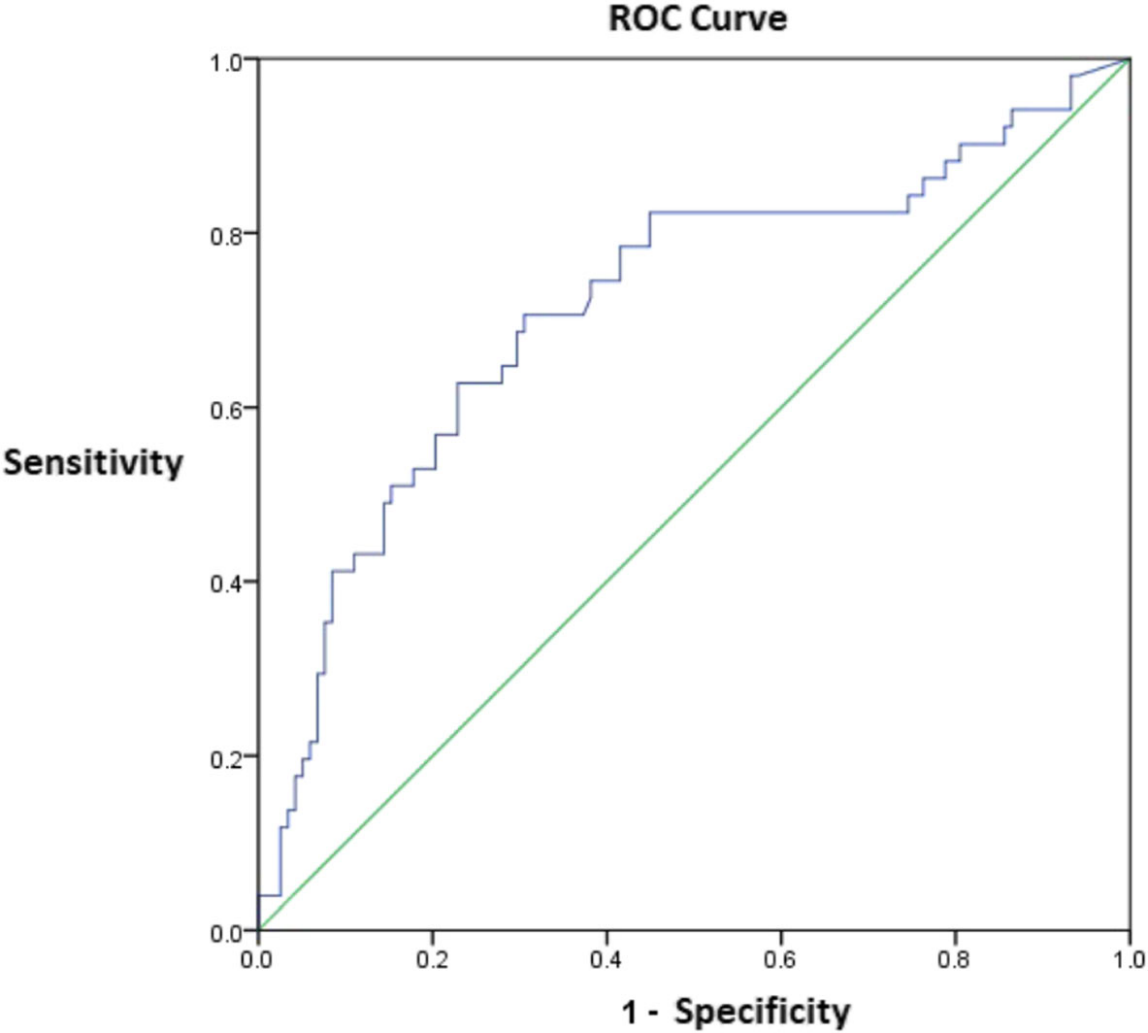
The ROC curve of E7 protein

When the ROC curve of E7 protein concentration was drawn, the AUC was 0.718 (95% CI 0.628–0.808) for the HPV16/18 E7 protein assay, and 8.270 was the optimal cut-off value (Fig. 2).

### The positive rate of E7 protein expression in CIN of different severities

E7 protein expression was considered positive if the test value was 8.27 or above. The positive rate was 26.6% (30/113) in pathologically normal/inflammation cases, including 18 cases of positive HPV16/18 test results and 7 cases with both negative cytologic test and HPV test results. The positive rate was 58.3% (14/24) in CIN1 cases, 70.6% (48/68) in CIN2+ cases and 67.4% (31/46) in CIN3+ cases. The results indicated that the positive rate of E7 protein expression increased with the severity of CIN: it was 32.1% in normal and CIN1 patients (the negative control group) and 70.6% in CIN2+ patients (the pathological positive group) (*P* < 0.001). (Table 1).

**Table 1:** The positive rate of E7 protein in CIN of different severities (n/N, %)

Pathology	N	Positive E7 cases (n)	Positive E7 rate (%)
Normal	113	30	26.6%
CIN1	24	14	58.3%
CIN2+	68	48	70.6%
CIN3+	46	31	67.4%
Total	205	92	44.9%

### Diagnostic efficacy of the E7 protein assay versus the HPV test

For CIN2+ detection, the E7 protein assay had a sensitivity, specificity, PPV, and NPV of 70.6, 67.9, 52.2, and 82.3%, respectively. For the HPV test, the values were 86.8, 44.5, 43.7, and 87.1%, respectively. The E7 protein assay was significantly less sensitive (*P* = 0.021) but was significantly more specific (*P* < 0.001). The combination of E7 protein assay and HPV test had a specificity and PPV of 78.1 and 58.3%, respectively, which were significantly higher than those of the HPV test alone (*P* < 0.001 and *P* = 0.045, respectively) (Table 2). For CIN3+ detection, the E7 protein assay had a sensitivity, specificity, PPV, and NPV of 67.4, 61.6, 33.7, and 86.7%, respectively. The combination of E7 protein assay and HPV test had a specificity of 72.3%, which was significantly higher than that of the HPV test alone (*P* < 0.001).

**Table 2:** Diagnostic efficacy of the E7 protein assay versus the HPV test (n/N, %)

	Sensitivity (%)	Specificity (%)	PPV (%)	NPV (%)
**CIN2+**				
**E7 protein**	70.6% (48/68)	67.9% (93/137)	52.2% (48/92)	82.3% (93/113)
**HPV**	86.8% (59/68)	44.5% (61/137)	43.7% (59/135)	87.1% (61/70)
**E7 protein + HPV**	61.8% (42/68)	78.1% (107/137)	58.3% (42/72)	80.4% (107/133)
**CIN3+**				
**E7 protein**	67.4% (31/46)	61.6% (98/159)	33.7% (31/92)	86.7% (98/113)
**HPV**	89.1% (41/46)	40.9% (65/159)	30.4% (41/135)	92.9% (65/70)
**E7 protein + HPV**	60.9% (28/46)	72.3% (115/159)	38.9% (28/72)	86.5% (115/133)

*Note*: CIN2 and CIN3 cases were considered as pathological positive; CIN1, normal, and TCT/Hr-HPV-negative cases were considered as negative, *PPV* positive predicative value, *NPV* negative predictive value

## Discussion

Hr-HPV is the causative pathogen of cervical cancer [17], The 2012 American Society for Colposcopy and Cervical Pathology guidelines state that patients positive for HPV16/18 should undergo colposcopy and biopsy [18], However, 90% of HPV infections are transient [3], so the positive predictive value of CIN2+ is only 11.4% in women with HPV16/18 and normal cytology [19]. This means that many patients without CIN are undergoing colposcopy, which increases the caseload of unnecessary colposcopies. Therefore, it is important to search for better auxiliary diagnostic markers in order to improve the accuracy rate.

As a protein product of Hr-HPV mRNA, E7 affects cell proliferation [10]. In this study, ROC analysis showed that the HPV16/18 E7 protein assay had an AUC of 0.718 for the diagnosis of CIN2+. For CIN2 and CIN3 cases, the sensitivity and the specificity were 70.6 and 67.9%, respectively. The specificity level was ideal for the diagnosis of CIN2 + .

This study showed that E7 protein level was correlated with the severity of CIN. With the cut-off expression value of 8.27, the positive rate was 26.6% in pathologically normal/inflamed cases, 58.3% in CIN1 cases, and 70.6% in CIN2/CIN3 cases, indicating that the positive rate increased with the severity of CIN. In CIN2 and CIN3 cases, the sensitivity was 70.6%, which was lower than that of the HPV test (86.8%), but the specificity was 67.9%, which was significantly higher than that of the HPV test (44.5%). With the combination of the E7 protein assay and the HPV test, the specificity was 78.1%, which was significantly higher than that of the HPV test alone (*P* < 0.001); the PPV was 58.3%, which was also higher than that of the HPV test alone. Besides, similar with CIN2+ cases, when combined the E7 protein assay with the HPV test in CIN3+ cases detection, the specificity was 72.3%, which was significantly higher than that of the HPV test alone (40.9%) (*P* < 0.001). If the HPV test is used alone for CIN screening, many patients with transient HPV infection (rather than precancerous lesions) will undergo colposcopy and biopsy, which causes anxiety and panic and increases healthcare costs. The combination of E7 protein assay and HPV test improves the specificity of CIN detection and reduces the colposcopy referral rate and invasive examination rate.

E7 protein expression was positive in 30 pathologically normal samples (26.6%), including 12 cases with negative HPV tests. This may be due to technical limitations. Moreover, 18 pathologically negative cases were positive on the HPV16/18 test. E7 protein level may increase before positive pathological findings or may be more sensitive than a pathological examination. In a previous study, E7 protein expression was positive in 14.3% of pathologically normal cases [20]. The 18 patients with positive HPV16/18 test should be followed up to further observe the relationship between E7 protein level and disease progression.

**Fig. 3:**
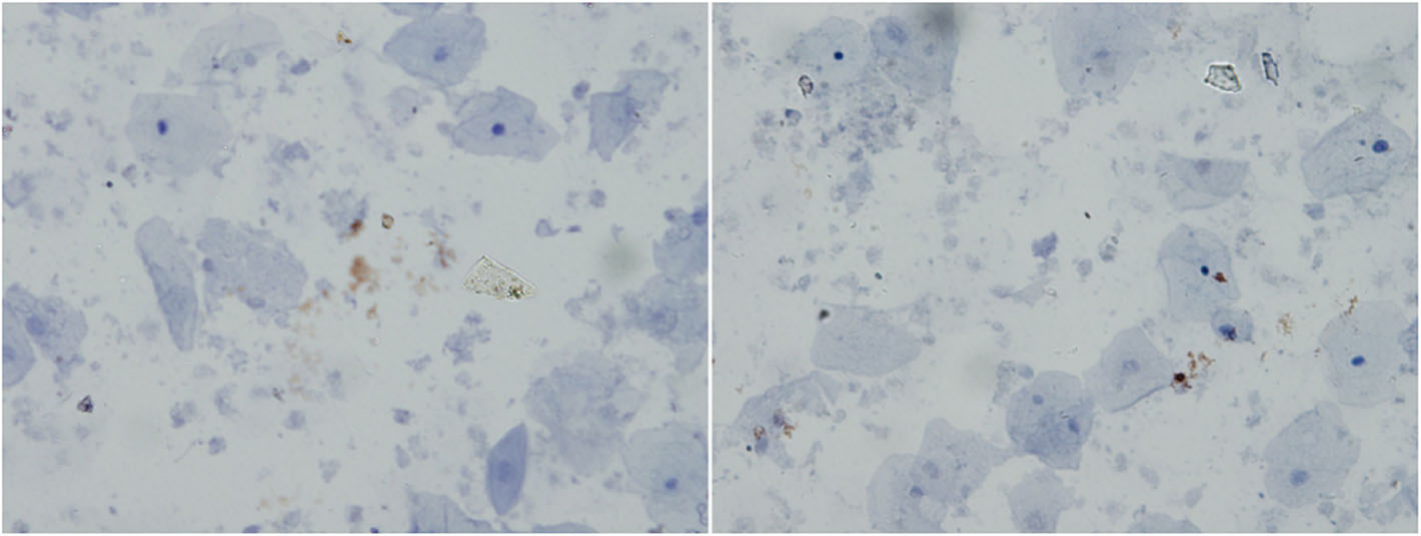
The immunocytochemistry staining of E7 protein in one of CIN3 samples

**Fig. 4:**
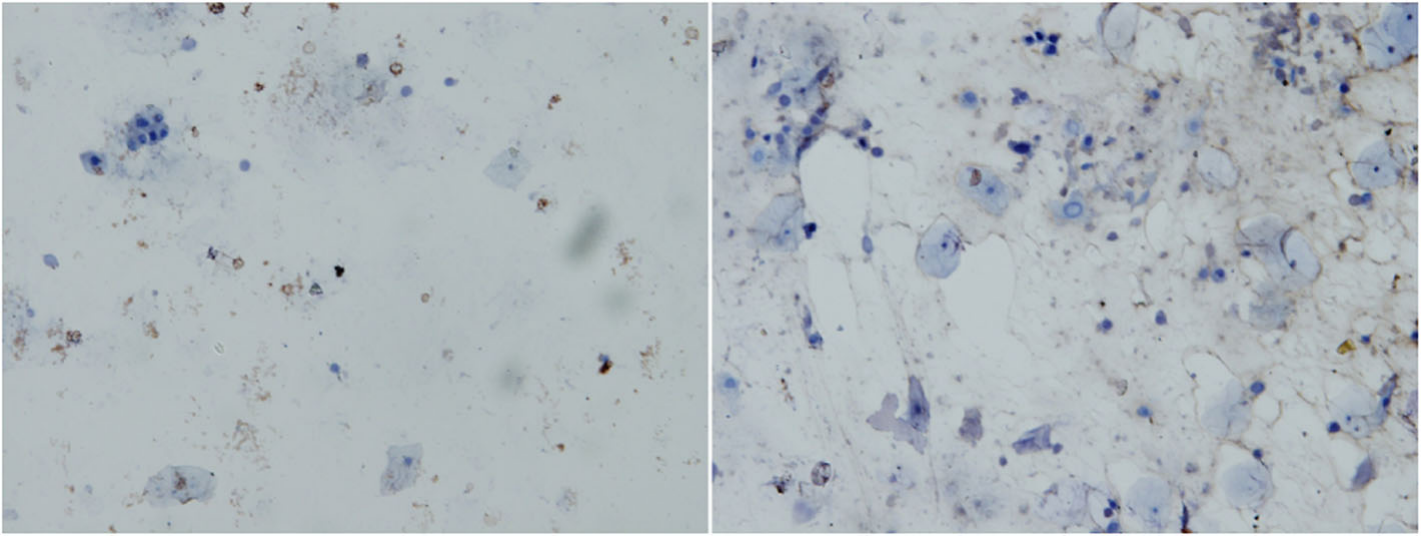
The immunocytochemistry staining of E7 protein in a cervical cancer sample

In this study, E7 protein expression was negative in 20 CIN2/CIN3 cases (20/68, 29.4%). The false-negative results may be related to sampling, laboratory procedures, or technical limitations. In our preliminary experiment, we analyzed the false-negative results of E7 protein in seven CIN2+ samples. The samples were tested using the BD Accuri C6 flow cytometer, and the results showed that each of the seven samples contained 16,000 to 760,000 cells. Moreover, streptavidin-peroxidase immunocytochemical staining detected E7 protein expression in two samples, however, the expression was detected in only a few cells. Specifically, one of the seven samples (CIN3) showed substantially intact cells, with low E7 protein expression in a few cells (brown particles in the nucleus and cytoplasm), as well as some cell fragmentation and protein release (Fig. 3). Another sample (cervical cancer) showed significant cell fragmentation with few normal cells and very low E7 protein expression due to cell fragmentation (Fig. 4). These data indicate that sampling has a significant impact on the E7 protein assay, suggesting that it is important to standardize the sampling procedures to ensure the optimal cell number and integrity for the E7 protein assay.

E6 and E7 are the most important oncogenes of HPV [21]. Studies have confirmed the role and value of the HPV E6 protein assay. In 2014, Qiao et al. [22] found that when diagnosing high-grade CIN, the sensitivity and specificity of the E6 protein assay were 42.8 and 94.3%, respectively, and in the study of Zhang et al. (2017) [23], the values were 44.8 and 93.5%, respectively. This study showed that the E7 protein assay has its own strength and limitations relative to the E6 assay, which was consistent with previous reports [20]. E7 protein is a promising auxiliary marker for the screening of CIN.

## Conclusions

In summary, this study shows that the combination of E7 protein assay and HPV test improves the diagnostic specificity and PPV for diagnosing CIN, which reduces the colposcopy referral rate and maybe one of promising strategies for minimizing regional variation in screening for CIN, and reduces healthcare costs. Large multicenter studies are needed to validate the results and continue the search for more effective screening tools for CIN.

## Data Availability

The datasets used and/or analyzed during the current study are available from the corresponding author on reasonable request.

## Abbreviations

CIN: Cervical intraepithelial neoplasia
Hr-HPV: High-risk human papillomavirus
PPV: Positive predictive value
NPV: Negative predictive value
WHO: World Health Organization
ASC-US: Atypical Squamous Cells of Undetermined Significance
ROC: Receiver operating characteristic
AUC: Area under the curve
