# Correction to: Analysis of the role of the human papillomavirus 16/18 E7 protein assay in screening for cervical intraepithelial neoplasia: a case control study

**DOI:** 10.1186/s12885-020-07547-0

**Published:** 2020-10-26

**Authors:** Linghua Kong, Xiaoping Xiao, Huiping Lou, Pengfei Liu, Shuhui Song, Moudu Liu, Tao Xu, Ying Zhang, Caijuan Li, Ruoli Guan, Yan Li, Xin Yu, Haiyuan Liu, Qingbo Fan, Honghui Shi, Lan Zhu

**Affiliations:** 1 Department of Obstetrics and Gynecology, Peking Union Medical College Hospital, Chinese Academy of Medical Sciences, Shuaifuyuan No. 1, Dongcheng District, Beijing, 100730 China; 2 Department of Health Sciences, Peking Union Medical College Hospital, Chinese Academy of Medical Sciences, Shuaifuyuan No. 1, Dongcheng District, Beijing, 100730 China; 3 FAMID Biomedical Technology (Tianjin) Co., Ltd., No. 4, Haitai Development 2nd Road, Binhai Gaoxin District, Tianjin, 300450 China; 4 Institute of Basic Medical Sciences, Peking Union Medical College, Chinese Academy of Medical Sciences, 5 Dong Dan San Tiao, Beijing, 100005 China

**Correction to: BMC Cancer 20, 999 (2020)**

**https://doi.org/10.1186/s12885-020-07483-z**

Following publication of the original article [1], the authors reported an error in affiliation 3. The correct affiliation 3 is: FAMID Biomedical Technology (Tianjin) Co., Ltd., No. 4, Haitai Development 2nd Road, Binhai Gaoxin District, Tianjin 300450, China.

The incorrect affiliation was also used in the method/E7 protein assay section.

The original article [1] has been corrected.
